# Intermittent fasting protects against food allergy in a murine model *via* regulating gut microbiota

**DOI:** 10.3389/fimmu.2023.1167562

**Published:** 2023-05-09

**Authors:** Ru-xue Ma, Jia-qian Hu, Wei Fu, Jian Zhong, Can Cao, Chang-chang Wang, Shi-quan Qi, Xiao-Lian Zhang, Guang-hui Liu, Ya-dong Gao

**Affiliations:** ^1^ Department of Allergology, Zhongnan Hospital of Wuhan University, Wuhan, China; ^2^ Department of Immunology, School of Basic Medical Sciences, Wuhan University, Wuhan, China; ^3^ Hubei Province Key Laboratory of Allergy and Immunology, Wuhan University, Wuhan, China

**Keywords:** food allergy, intermittent fasting, gut microbiota, type 2 inflammation, intestinal epithelial barrier

## Abstract

**Background:**

The prevalence of food allergy (FA) is increasing. Decreases in the diversity of gut microbiota may contribute to the pathogenesis of FA by regulating IgE production of B cells. Intermittent fasting (IF) is a popular diet with the potential to regulate glucose metabolism, boosting immune memory and optimizing gut microbiota. The potential effect of long-term IF on the prevention and treatment of FA is still unknown.

**Methods:**

Two IF protocols (16 h fasting/8 h feeding and 24 h fasting/24 h feeding) were conducted on mice for 56 days, while the control mice were free to intake food (free diet group, FrD). To construct the FA model, all mice were sensitized and intragastrical challenged with ovalbumin (OVA) during the second half of IF (day 28 to day 56). Rectal temperature reduction and diarrhea were recorded to evaluate the symptoms of FA. Levels of serum IgE, IgG1, Th1/Th2 cytokines, mRNA expression of spleen T cell related transcriptional factors, and cytokines were examined. H&E, immunofluorescence, and toluidine blue staining were used to assess the structural changes of ileum villi. The composition and abundance of gut microbiota were analyzed by 16srRNA sequencing in cecum feces.

**Results:**

The diarrhea score and rectal temperature reduction were lower in the two fasting groups compared to the FrD groups. Fasting was associated with lower levels of serum OVA-sIgE, OVA-sIgG1, interleukin (IL)-4 and IL-5, and mRNA expression of IL-4, IL-5, and IL-10 in the spleen. While no significant association was observed in interferon (IFN)-γ, tumor necrosis factor (TNF)-α, IL-6, IL-2 levels. Less mast cell infiltration in ileum was observed in the 16h/8h fasting group compared to the FrD group. ZO-1 expression in the ileum of the two fasting groups was higher in IF mice. The 24h/24h fasting reshaped the gut microbiota, with a higher abundance of *Alistipes* and *Rikenellaceae* strains compared to the other groups.

**Conclusion:**

In an OVA-induced mice FA model, long-term IF may attenuate FA by reducing Th2 inflammation, maintaining the integrity of the intestinal epithelial barrier, and preventing gut dysbiosis.

## 1 Introduction

Food allergy (FA) is an abnormal immune reaction triggered by normally innocuous food protein allergens (1). The prevalence of FA is increasing worldwide, especially in children. Rigorous avoidance of culprit foods is still the primary strategy to prevent FA but involves a risk of malnutrition in children (2, 3). FA is the most common cause of anaphylaxis and can thus be potentially life-threatening. The atopic march may begin in infancy with atopic dermatitis and food allergy, and in certain cases can develop into allergic asthma and allergic rhinitis in childhood (4, 5), which is caused by multiple immunological pathways, including allergen exposure, environmental pollutants, skin barrier dysfunction, type 2 inflammation, and oxidative stress (5). More importantly, FA is also associated with a greater risk and severity of asthma in both adults and children (6–8). Thus, early detection and proper treatment of FA are essential to prevent or stop the atopic march (9).

In recent years, environmental changes have been recognized as the main factor driving the development of FA (10). Environment changes may decrease the diversity of gut microbiota, which is a key factor in the pathogenesis of FA (11). Gut microbiota dysbiosis occurs earlier than FA (12, 13). Increasing the richness of the microbiota can inhibit the production of IgE by B cells (14). In addition, gut microbial species such as *Clostridium*, *Lactobacillus (* 15), and *Rhamnosus GG (* 16) were shown to be protective against FA (14). Changes in gut microbiota composition were shown to reduce the susceptibility to FA in mice model (14–17). Moreover, the transfer of fecal microbiome from healthy infants can protect germ-free mice from cow’s milk allergy evoked by β-lactoglobulin (12).

Having normal gut microbiota in the early life of mammals can promote immune tolerance (18). Mechanistically, polysaccharide A produced by *Bacteroides fragilis* induces the development of regulatory T cells (Tregs) through Toll-like receptor 2 (TLR2). *Clostridium cluster* therapy stimulates Tregs through the MyD88/retinoic acid receptor-related orphan receptors (RORγt) pathway to suppress FA (19). Other unclassified commensal bacteria were also shown to induce the expression of RORγt in colonic Tregs. Short-chain fatty acids (SCFAs) secreted by *Firmicutes*, such as butyrate, can inhibit histone deacetylases, promote the expression of transcription factor Foxp3, and enhance the suppressive function of Tregs (20). Probiotics can promote Th1 immune response rather than th2 (21–24). In the presence of *Bifidobacterium longum* and *B. animalis strains*, human peripheral blood T and NK cells can increase the production of IFN-γ and TGF-β (22). *Lactobacillus* also promotes the production of Th1 cytokine IFN-γ by inducing IL-12 and IL-18 expression in leukocytes (25–27). By contrast, colonization of harmful bacteria enhances Th2 inflammation. A higher fecal burden of *Escherichia coli* and *Clostridium difficile* and a higher *Clostridium difficile* to *Bifidobacterium* ratio in infants are associated with allergic disease (28–31). Germ-free or antibiotic-treated mice have increased type 2 immune responses and susceptibility to allergy, suggesting a regulatory effect of normal gut microbes on Th2 inflammation (32).

The factors influencing the composition of commensal microbes, such as delivery mode, feeding patterns, and the use of antibiotics, impact also the susceptibility of FA. It was found that elective cesarean section was associated with a higher incidence of FA compared to vaginal delivery in the offspring of mothers without atopic diseases (33). Babies born vaginally acquired microbiota (in the skin, oral cavity, and nasopharyngeal) similar to the mother’s vaginal microbiome (mainly *Lactobacilli* and *Prevotella*), whereas babies born by cesarean section had microbiota more similar to the mother’s skin surface (34). Breastfeeding directly transfers the human milk microbiome, including *Lactobacilli* and *Bifidobacterium* to babies. Breast milk is rich in oligosaccharides, sIgA, glycopeptides, tryptophan metabolites, lipids, soluble TLR2/TLR4 and their co-receptors, antimicrobial proteins and peptides which can indirectly guide the growth of microorganisms (20). Lactoferrin in breast milk acts as a protective protein against the production of reactive oxygen species, phagocytic activity, and Toll-like receptor expression, and thus may prevent the development of allergies to some degree (35). Breastfeeding is currently recommended as primary prevention for allergic diseases, including FA (36), and non-breastfeeding or breastfeeding for short durations is associated with higher asthma risk in childhood (37).

Humans adapted to intermittent fasting (IF) due to the difficulty of obtaining food in ancient times. In modern times, IF is adopted by many people for losing weight, improving metabolic syndrome, and preventing cardiovascular and cerebrovascular diseases (38). In addition, IF can decrease the risk of diabetes by reducing the deposit of belly fat and the production of free radicals, improving glucose metabolism (39, 40). IF can lower blood pressure, stabilize heart rate, and improve heart rate variability and autonomic balance (41). Moreover, IF has a regulatory effect on immunity such as boosting immune memory and suppressing inflammation (42). Time-restricted feeding restores normal circadian rhythms and extends lifespan further in a Drosophila model (43).

Importantly, IF can increase the diversity of gut microbiota and improve the richness of *Lactobacillus*, *Prevotella*, and *Bacteroidaceae (* 44). In Muslim countries with Ramadan practices, such as Kuwait, Lebanon, and Turkey, the incidence of FA appears to be lower than in European countries (45), suggesting a potential role of IF in the prevention of FA. A previous study in a mice model showed that temporary fasting prior to immunization with OVA was associated with exacerbated FA symptoms such as diarrhea and impaired immune responses such as induction of intestinal sIgA, plasma OVA-specific IgM and IgG (46). Whether long-term intermittent fasting has a preventive or therapeutic effect on FA is still unknown.

In the current study, we investigated the effect of long-term fasting on FA in an OVA-sensitized and challenged murine model of FA. In addition, the impact of long-term fasting on the richness, diversity, and components of gut microbiota was also determined.

## 2 Materials and methods

### 2.1 Mouse husbandry

Thirty female Balb/c mice (6-8 weeks old, provided by Wuhan Wanqian Jiaxing Biotechnology Co., Ltd) were housed in an SPF barrier environment on a 12-hour light/dark cycle at 20-24°C. Mice had free access to sterile drinking water. Poplar bedding material was used to prevent mice from chewing on padding and changed weekly. All experimental processes were approved by the Animal Ethics Committee of Wuhan University (IACUC NO. ZN2022070).

### 2.2 Protocols for intermittent fasting

After acclimation for one week, mice were divided into a free diet group (FrD, food was available at all times) and two fasting groups (47) (16h: 8h fasting (16/8F), fasted for 16 hours and fed for 8 hours (9:00 am- 5:00 pm) every day; 24h:24h fasting (24/24F), alternatively fed and fasted for 24 hours, food was supplied at 9: 00 am in every other day). The food did not contain any egg or milk ingredients.

### 2.3 Murine model of FA

**Figure 1:**
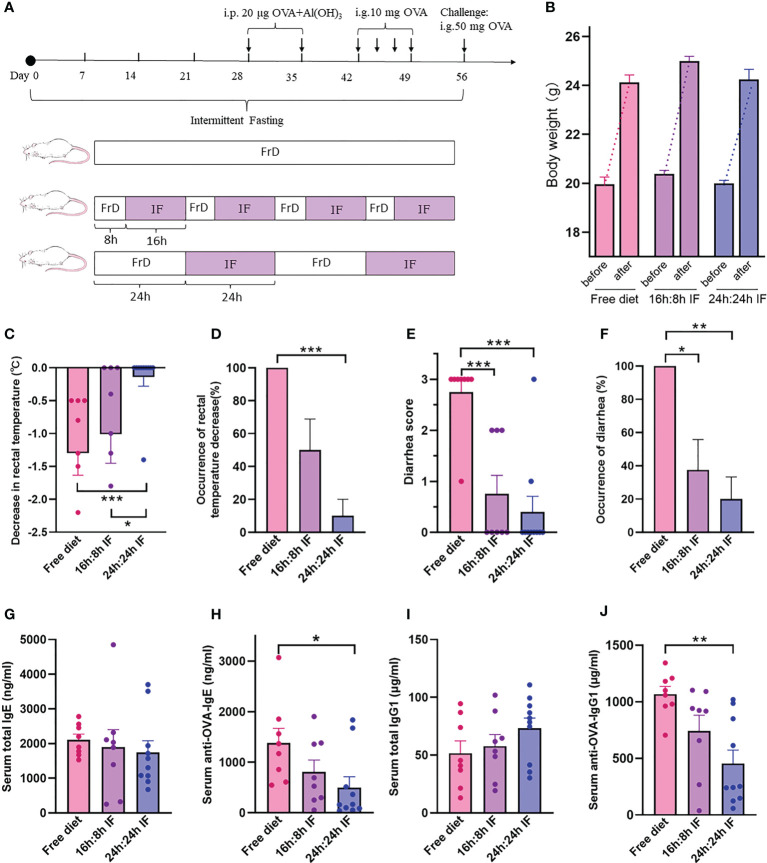
The effects of intermittent fasting on food allergy. **(A)** Experimental design. Free Diet: FrD; Intermittent Fasting: IF. **(B)** Body weight before and after the experiment. **(C)** Decrease in rectal temperature after ovalbumin antigen challenge. **(D)** Proportion of rectal temperature decrease. **(E)** Diarrhea score after challenge, from 0 to 3, solid feces=0; funicular form feces=1; slurry feces=2; watery feces=3. **(F)** Incidence rate of diarrhea. **(G)** Serum total IgE levels. **(H)** Serum anti-OVA-IgE levels. **(I)** Serum total IgG1 levels. **(J)** Serum anti-OVA-IgG1 levels. **(B–J)**: n=8,8,10 respectively in FrD group, 16h: 8h IF group, 24h: 24h IF group. Error bars represent S.E.M. The significance was analyzed by Mann-Whitney test in **(B–F)**, and two independent-sample t-test in **(G–J)**. *p < 0.05; **p < 0.01; ***p < 0.001.

After 4 weeks of IF, mice were sensitized by intraperitoneal injection of 50 μg OVA (Sigma-Aldrich) mixed with aluminum hydroxide gel (Thermo Fisher Scientific) on days 28 and 35. From day 42 to 49, mice were challenged by intragastric administration of 10 mg OVA dissolved in 0.2 mL PBS every other day. On the last day of the protocol (day 56), mice were challenged by intragastric infusion of 50 mg OVA (48) (**Figure 1A**). Fresh fecal samples were collected in the sterile test tubes on day 28 and 50 and stored at -80°C for later use. The sera were separated from eyeball blood 3 hours after the OVA challenge before mice were sacrificed, and stored at -20°C. Spleens, terminal ileum, and feces in the cecum were collected after sacrificing mice.

### 2.4 Evaluation of food allergy symptoms

Rectal temperature was measured 15 min before and at intervals of 15 min, 30 min, and 60 min after OVA challenge. A deeper temperature decrease indicates a more severe food allergy. The rectal temperature at 15 min before the challenge was set as the baseline, and a 0.5°C or more decrease in rectal temperature was the threshold of FA symptoms. Diarrhea scores were evaluated according to the feces traits: 0, solid feces; 1, funicular form feces; 2, slurry feces; 3, watery feces (48).

### 2.5 Immunoglobin and cytokine assay

Serum levels of total IgE, IgG1, OVA-specific IgE (sIgE), and OVA-specific IgG1 (sIgG1) were determined using the commercial ELISA kits (Cayman Chemical Company) according to the instructions. Serum IL-5, IL-13, IL-2, IL-6, IL-10, IFN-γ, TNF-α, IL-4 level were determined by Cytometric Bead Array (CBA, BioLegend, Inc.).

### 2.6 Histology and immunofluorescence examination

Mice ilea were fixed in 4% paraformaldehyde for 24 h at room temperature, dehydrated, and embedded into paraffin. Ileum tissues were sectioned into 5 μm thickness slices and stained with hematoxylin and eosin (HE), and toluidine blue. A 400× field of vision was selected randomly in each tissue sample for mast cell counting. For immunofluorescence, the slides were incubated overnight at 4°C with rabbit-anti-mouse ZO-1 primary antibody (1:350, Proteintech Group, Inc.), and then incubated for 1 h at 37°C with CoraLite488-conjugated goat-anti-rabbit IgG (H+L) (1:100, Proteintech Group, Inc.). In each sample, nine small squares on the intestinal villi were selected randomly and the average area with ZO-1 fluorescence was calculated.

### 2.7 RT-qPCR

Dissected spleens were dipped in RNAwait (Beijing Solarbio Science & Technology Co., Ltd.) overnight at 4°C. Total RNA was extracted with TRIpure reagent (Aidlab Biotechnologies Co., Ltd), and the purity and concentration were assessed by a NanoDrop one spectrophotometer (Thermo Fisher Scientific). RNA was then reversely transcribed into cDNA with Oligo(dt) primers and M-MLV RT (TOYOBO Japan Co., Ltd.). The primers of T-bet, GATA3, FoxP3, RORγT, IL-4, IL-5, IL-10, IL-13, IFN-γ, and TGF-β (Tgfb1) (**Table S1**) were referenced from PrimerBank (https://pga.mgh.harvard.edu/primerbank) and synthesized by Tsingke Biotechnology Co., Ltd. GAPDH was used as a loading control. SYBR green reagent (Vazyme Biotech Co., Ltd) was used to measure the cDNA according to the manufacturer’s instructions. Relative expression of mRNA was measured using the 2^–ΔΔCt^ method.

### 2.8 Flow cytometry

Single cell suspensions of the spleen were prepared by passing the cells through 70μm cell strainers, then the erythrocytes were lysed by incubation with red cell lysis buffer. The separated leukocytes were incubated in PRMI-1640 medium containing serum in the presence of concanavalin A (as positive control) (2 μl/ml, Thermo Fisher Scientific) or OVA (100 μg/ml) for three days. Leukocytes were then stained with the mouse Treg Flow™ Kit (BioLegend, Inc.) according to the manufacturer’s instructions, and the proportion of CD4^+^CD25^+^Foxp3^+^ Tregs were detected by flow cytometry.

### 2.9 16s rRNA sequencing

16s rRNA sequencing was used to assess the microbiota composition in cecum feces. Gut microbiota DNA was extracted and analyzed with agarose gel electrophoresis to determine the purity and concentration. The DNA samples were then diluted to 1 ng/μl and used for PCR to amplify the 16S V4 region with Phusion^®^ High-Fidelity PCR Master Mix (New England Biolabs Inc.). TruSeq^®^ DNA PCR-Free Sample Preparation Kit (Illumina) was used for library construction, and NovaSeq6000 (Illumina) was used for on-machine sequencing.

The relative abundance column accumulation was plotted of the top 10 species with maximum abundance at the phylum level for each sample or group. The heatmap of species abundance clustering was plotted based on species annotation and abundance information at the genus level, while the top 35 genera were clustered. A certain amount of sequencing data were extracted randomly from the sample, and the number of species they represent (i.e., OTUs) was calculated. The rarefaction curve was constructed by the amount of sequencing data and the corresponding number of species. The OTUs were sorted descending according to the relative abundance as an ordinal number for abscissa. The rank abundance curve was plotted by connecting the corresponding points (49). The species accumulation boxplot was used to describe the increase in species diversity and sample size, investigate the species composition, and predict species abundance. The similarity percentage, a decomposition of the Bray-Curtis dissimilarity, was analyzed to quantify the magnitude of the contribution of each species to the difference between the two groups. The bacterial community structure was analyzed to identify species with significant differences in abundance and magnitude among groups. For the Meta-Stat method, a hypothesis test was performed on species abundance between groups to obtain a p-value, which was then corrected by Benjamini-Hochberg false discovery rate to get the q-value (50). Species with significant differences were selected according to the q value, and the box plots of the abundance were drawn. The phylogenetic tree was drawn by the representative sequences of the top 100 genera, which were obtained by multiple sequence alignment.

### 2.10 Statistical analysis

All data were expressed as mean ± SEM. Statistical differences between groups were analyzed using Student’s t-test on Gaussian distribution or Mann-Whitney Wilcoxon on non-Gaussian distribution. p values < 0.05 were considered statistically significant.

## 3 Results

### 3.1 IF reduced the symptoms of FA

The average decrease of rectal temperature was 1.3°C, 1.0°C, and 0.14°C respectively in FrD, 16/8F, and 24/24F groups. Rectal temperature decrease was observed in 100% of the FrD group, 50% of the 16/8F group, and only 10% of 24/24F. Both the averaged degree and incidence rate of rectal temperature decrease were significantly higher in the FrD group than in the 24/24F group (p < 0.001, **Figures 1C, D**). Diarrhea was observed in 100% of the FrD group, which was significantly higher than that in the 16/8F group (37.5%, p < 0.05) and in the 24/24F group (20%, p < 0.01) (**Figure 1F**). The averaged diarrhea score was 2.75 in FrD group, which was significantly higher than that in the 16/8F (0.75, p < 0.001) and 24/24F groups (0.4, p < 0.001) respectively (**Figure 1E**).

The weight gain of fasted mice during growth was not different from that of the normal group, which indicates that IF will not induce malnutrition in these mice (**Figure 1B**).

The serum level of total IgE tended to decrease with fasting degree, i.e., FrD >16/8 F > 24/24F, but without statistical significance (**Figure 1G**). The serum levels of the OVA-sIgE were significantly lower in the 24/24F group (498.7 ng/ml) compared to the FrD group (1379.9 ng/ml, p < 0.05) (**Figure 1H**). The serum total IgG1 level tended to increase with the fasting degree but without statistical significance (**Figure 1I**). By contrast, the serum levels of OVA-sIgG1 tended to decrease with the fasting degree. The serum OVA-sIgG1 was 453.7μg/ml in the 24/24F group, which was significantly lower compared with the FrD group (1068.2 μg/ml, p < 0.01) (**Figure 1J**).

These data suggested that IF significantly attenuated FA symptoms and OVA-sIgE levels in the mice model. The 24h: 24h fasting protocol seems to be more efficient at relieving FA compared with the 16h: 8h fasting protocol.

### 3.2 Effect of IF on serum levels of Th1 and Th2 cytokines

**Figure 2:**
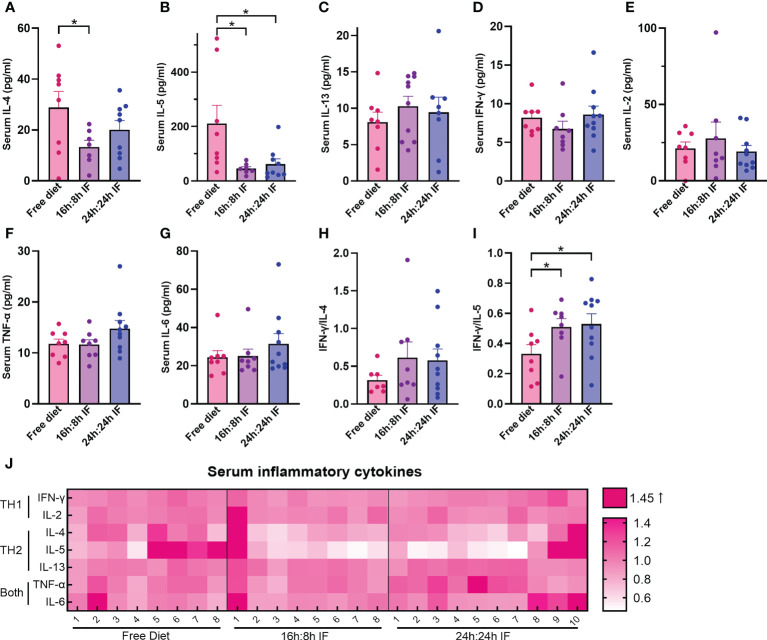
Serum levels of Th1 and Th2 cytokines. **(A)** IL-4. **(B)** IL-5. **(C)** IL-13. **(D)** IFN-γ. **(E)** IL-2. **(F)** TNF-α. **(G)** IL-6. **(H)** IFN-γ/IL-4. **(I)** IFN-γ/IL-5. **(J)** Heat map of above serum inflammatory factors, normalized CBA-MFI was used. CBA-MFI: mean fluorescence intensity of beads in the system (each cytokine per mouse) tested by cytometric bead array method. **(A–I)**: n=8,8,10 respectively in FrD group, 16h: 8h IF group, 24h: 24h IF group. Error bars represent S.E.M. The significance was analyzed by two independent-sample t-test in **(A, C–I)**, and Mann-Whitney test in **(B)**. *p < 0.05.

We then assessed the immune responses of IF by testing the serum levels of Th1 and Th2 cytokines IFN-γ, IL-2, TNF-α, IL-6, IL-4, IL-5, and IL-13. IL-4 was 28.9 pg/ml in the FrD group, significantly higher than that in the 16/8F group (13.3 pg/ml, p < 0.05) (**Figure 2A**). IL-5 was 210.6 pg/ml in the FrD group, which was significantly higher than that in 16/8F (45.4 pg/ml, p < 0.05) and 24/24F group (61.9 pg/ml, p < 0.05) (**Figure 2B**). Serum IL-13, IFN-γ, IL-2, and IL-6 levels were not different among the three groups (**Figures 2C–G**). The surrogate of Th1/Th2 bias, the IFN-γ/IL-4 ratio was higher in both fasting groups but without statistical significance, whereas the IFN-γ/IL-5 ratio was significantly higher in both fasting groups compared to the FrD group (p < 0.05) (**Figures 2H, I**).

Heat map plotting shows that the serum levels of Th2 cytokines were lower in both fasting groups than in the FrD group, while the serum levels of Th1 cytokines were close among the three groups (**Figure 2J**). These data suggested that IF is associated with lower type 2 inflammation.

### 3.3 Effect of IF on the differentiation of spleen T cells

**Figure 3:**
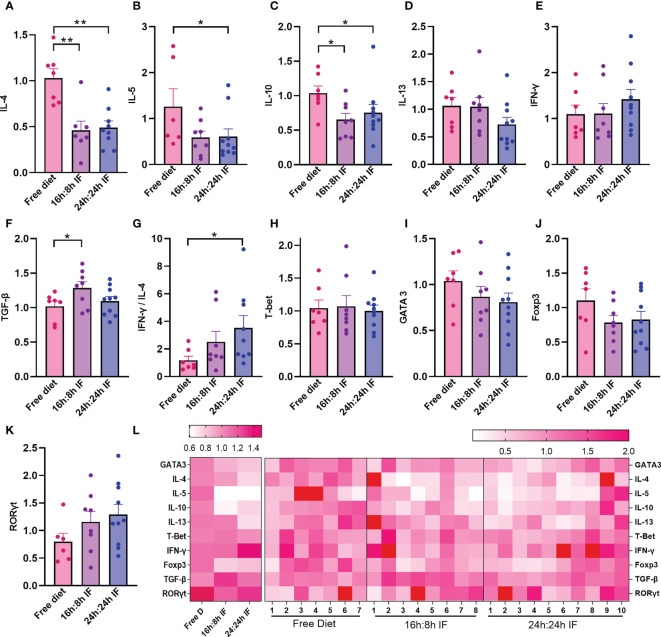
mRNA expression levels of cytokines and transcriptional factors in spleen. **(A)** IL-4. **(B)** IL-5. **(C)** IL-10. **(D)** IL-13. **(E)** IFN-γ. **(F)** TGF-β. **(G)** IFN-γ/IL-4 ratio. **(H)** T-bet. **(I)** Gata3. **(J)** Foxp3. **(K)** RORγt. **(L)** Heat map of the above mRNA expression levels. **(A–K)**: n=7,8,10 respectively in FrD group, 16h: 8h IF group, 24h: 24h IF group. Error bars represent S.E.M. The significance was analyzed by two independent-sample t-test in **(A, C–K)**, and Mann-Whitney test in **(B)**, *p < 0.05, **p < 0.01.

We then investigated Th1, Th2, Th17, and Tregs lymphocytes subgroups and their differentiation in the spleen. The mRNA expression levels of IL-4 in the FrD group were significantly higher than that in the 16/8F and 24/24F groups (p < 0.01). Similar results were found for IL-10 (p < 0.05) (**Figures 3A, C**). The mRNA expression of IL-5 was significantly higher in the FrD group than that in the 24/24F group (p < 0.05) (**Figure 3B**). The mRNA expression level of IFN-γ tended to increase, while IL-13 tended to decrease with fasting degree but without statistical significance (**Figures 3D, E**). The expression of TGF-β 1 was significantly higher in the 16/8F group compared to the FrD group (p < 0.05) (**Figure 3F**). The mRNA expression ratio of IFN-γ to IL-4 was calculated to indicate Th1/Th2 imbalance, and showed that Th1/Th2 ratio tended to increase with fasting degree. The IFN-γ/IL-4 ratio of the 24/24F group was significantly higher than that in the FrD group (p < 0.05) (**Figure 3G**). The mRNA expression of cytokine in the spleen was consistent with serum cytokine levels.

The mRNA expression of transcriptional factors for Th1, Th2, Tregs, and Th17 cells, T-bet, GATA3, Foxp3, and RORγt, were not significantly different among the three groups (**Figures 3H–K**). The heat map plotting with mRNA expression levels showed that IF significantly reduced Th2 inflammation compared to the FrD group (**Figure 3L**).

### 3.4 Effect of IF on spleen CD4^+^CD25^+^Foxp3^+^ Tregs

When stimulated with ConA, the proportion of CD4^+^CD25^+^Foxp3^+^ Tregs in *in vitro* cultured spleen cells tended to decrease with the degree of fasting: FrD group > 16h:8h IF group > 24h: 24h IF group (**Figures S2A, B**). The opposite trend was observed while stimulated with OVA, the proportion of Tregs was highest in the 24h: 24h IF group and lowest in the FrD group (**Figures S2C, D**). It seems that more Tregs were expanded in response to OVA stimulation in the fasting groups than in the FrD group.

### 3.5 Intestinal barrier was preserved by IF

**Figure 4:**
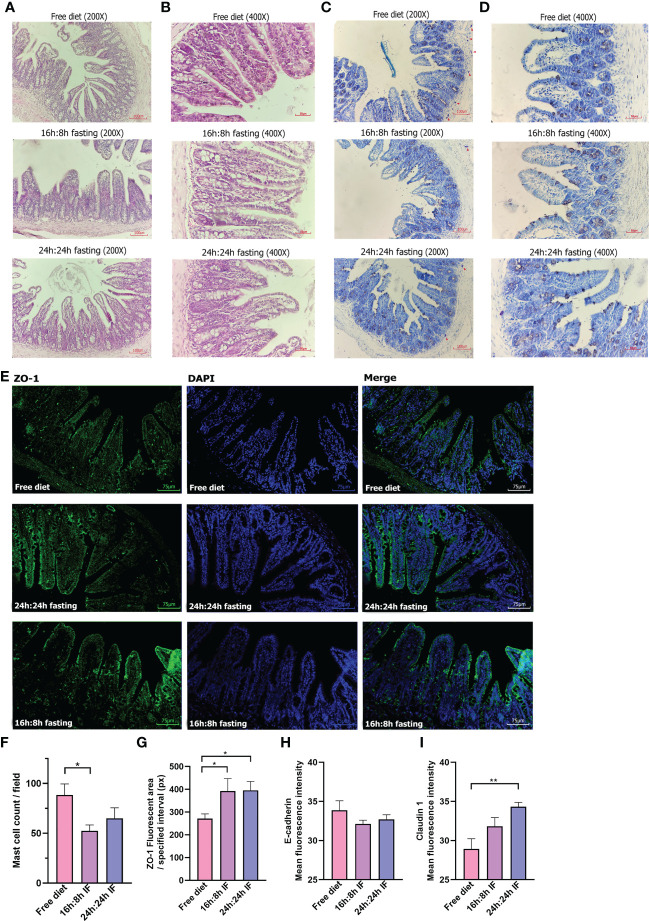
Pathology of the terminal ileum. **(A)** HE stain, 200×. **(B)** HE stain, 400×. **(C)** Toluidine blue stain, 200×. **(D)** Toluidine blue stain, 400×. Red triangles, label mast cells. **(E)** ZO-1 immunofluorescence, 200×. **(F)** Mast cell counting of each field. **(G)** ZO-1 fluorescent area. Nine squares located in the intestinal villi of each sample were randomly selected, then the fluorescence area was measured and averaged. **(H)** E-cadherin mean fluorescence intensity. **(I)** Claudin 1 mean fluorescence intensity. **(F, G)**: n=4 per group. **(H, I)**: n=5 per group. Error bars represent S.E.M. The significance was analyzed by two independent-sample t-test in **(F, H, I)** and Mann-Whitney test in **(G)**, *p < 0.05. **p < 0.01.

OVA-induced impairment of gastrointestinal mucosa was observed and HE staining showed that the intestinal villi of the terminal ileum were messy in all three groups (**Figures 4A, B**) without significant difference. Toluidine blue staining showed blue-purple mast cells scattered in the intestinal villi and submucosa, and degranulated mast cells had lighter staining (**Figures 4C, D**). The number of mast cells was significantly lower in the 16/8F group compared to the FrD group (p < 0.05, **Figure 4F**). Immunofluorescence staining showed that the tight junction (TJ) protein ZO-1 had a lower expression in intestinal epithelial of FrD group compared to both fasting groups (p < 0.05) (**Figures 4E, G**). And the expression of claudin-1 in the FrD group was significantly lower than in the 24h: 24h IF group (p < 0.01) (**Figure 4I** and **Figure S3B**), whereas the expression of E-cadherin did not show any difference among the three groups (**Figure 4H** and **Figure S3A**). These results suggest that fasting may preserve the integrity of the intestinal epithelial barrier.

### 3.6 Effect of IF on gut microbiota

**Figure 5:**
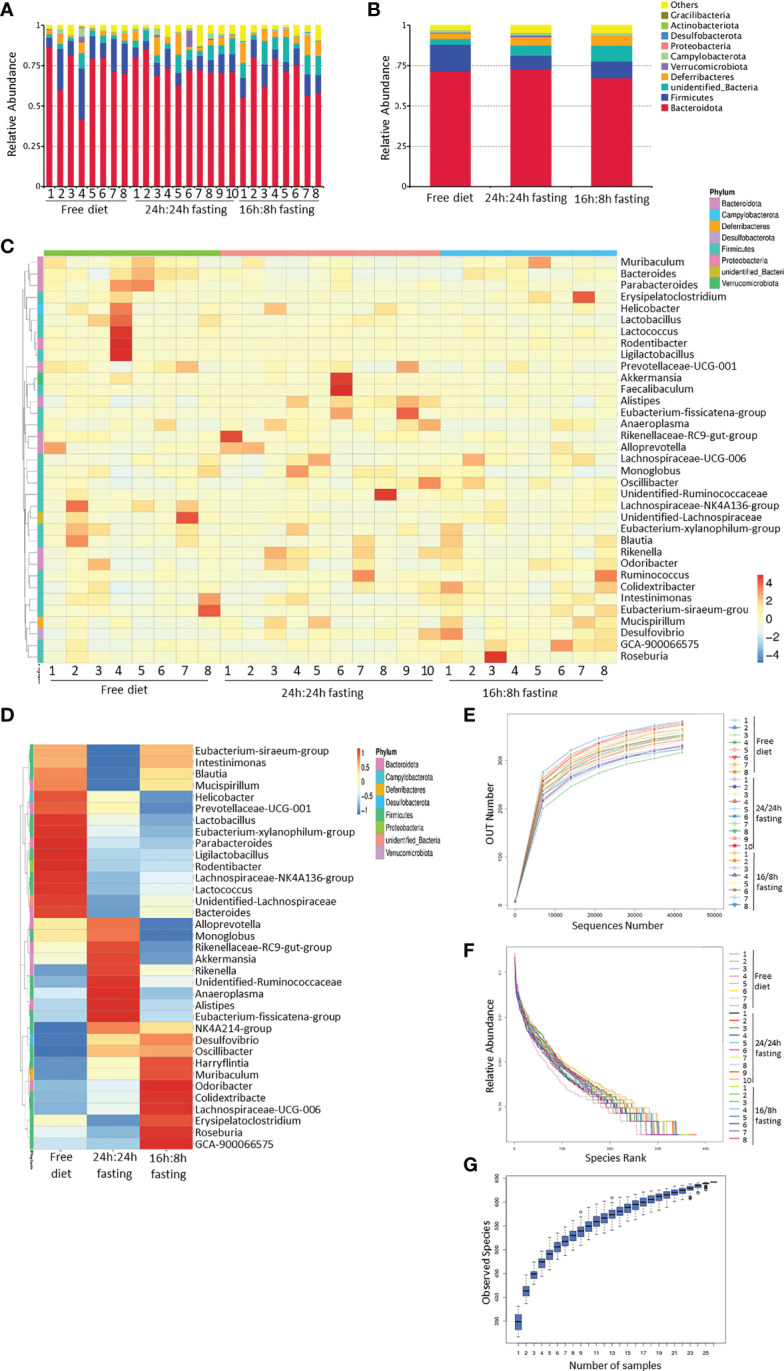
16s rRNA sequencing of gut microbiota, whole bacteria analysis. **(A, B)** Column summation of relative abundance of species at the phylum level. The top 10 species in abundance were selected, ‘Others’ represents the sum of the relative abundances of all the other phyla except for these 10 phyla. **(A)** in each mouse; B, in each group. **(C, D)** Heatmap of species abundance clustering. The top 35 genera in abundance were selected. **(C)** each mouse; **(D)** each group. **(E)** Rarefaction curves of sample diversity. **(F)** Rank Abundance of sample diversity. **(G)** Species accumulation boxplot. All the Figures n=8,8,10 respectively in the FrD group, 16h: 8h IF group, 24h: 24h IF group.

Cecum feces were analyzed by 16s rRNA gene sequencing among three groups. The relative abundance column accumulation plot showed that IF resulted in a decrease in the abundance of *Firmicutes* compared to the FrD group, while the unidentified bacteria and “other” bacteria had the opposite trend (**Figures 5A, B**).

The contribution of outliers must be considered and excluded in the heatmap of species abundance clustering arranged by groups. The high abundance of *Ligilactobacillus*, *Rodentibacter*, *Lactococcus*, *Lactobacillus*, and *Helicobacter* in the FrD group were primarily due to the outlier ‘mouse 4’. The enrichment of *Akkermansia* and *Faecalibaculum* in the 24/24F group was mainly due to the outlier ‘mouse 6’. To sum up, the richness of *Alistipes*, *Eubacterium-Fissicatena-group*, *Anaeroplasma*, *Rikenellaceae-RC9-gut-group*, *Alloprevotella*, and *Monoglobus* were higher in the 24/24F group than other two groups, only some of those bacteria showed statistically different trends, which will be noted later. The richness of *Odoribacter*, *Colidextribacter*, and *Lachnospiraceae-UCG-006* was highest in the 16h: 8h IF group, lowest in the FrD group. (**Figures 5C, D**). The rarefaction curve, rank abundance curve, and species accumulation boxplot showed the adequacy of sample size in three groups, and the sequencing was reasonable (**Figures 5E–G**).

The differences in gut microbiota biodiversity among the three groups were reflected by Alpha diversity. The ACE diversity tended to be higher in the 24h: 24h IF group when compared to the other two groups, but a significant difference was detected only when compared to the 16h: 8h IF group (**Figure S4A**). The unweighted principal coordinates analysis (PCoA) revealed the community differences among the three groups. FrD group and 16h: 8h IF group appeared to have more divergent gut microbiota, while the 24h: 24h group had more overlap regions with the other two groups (**Figure S4D**).

**Figure 6:**
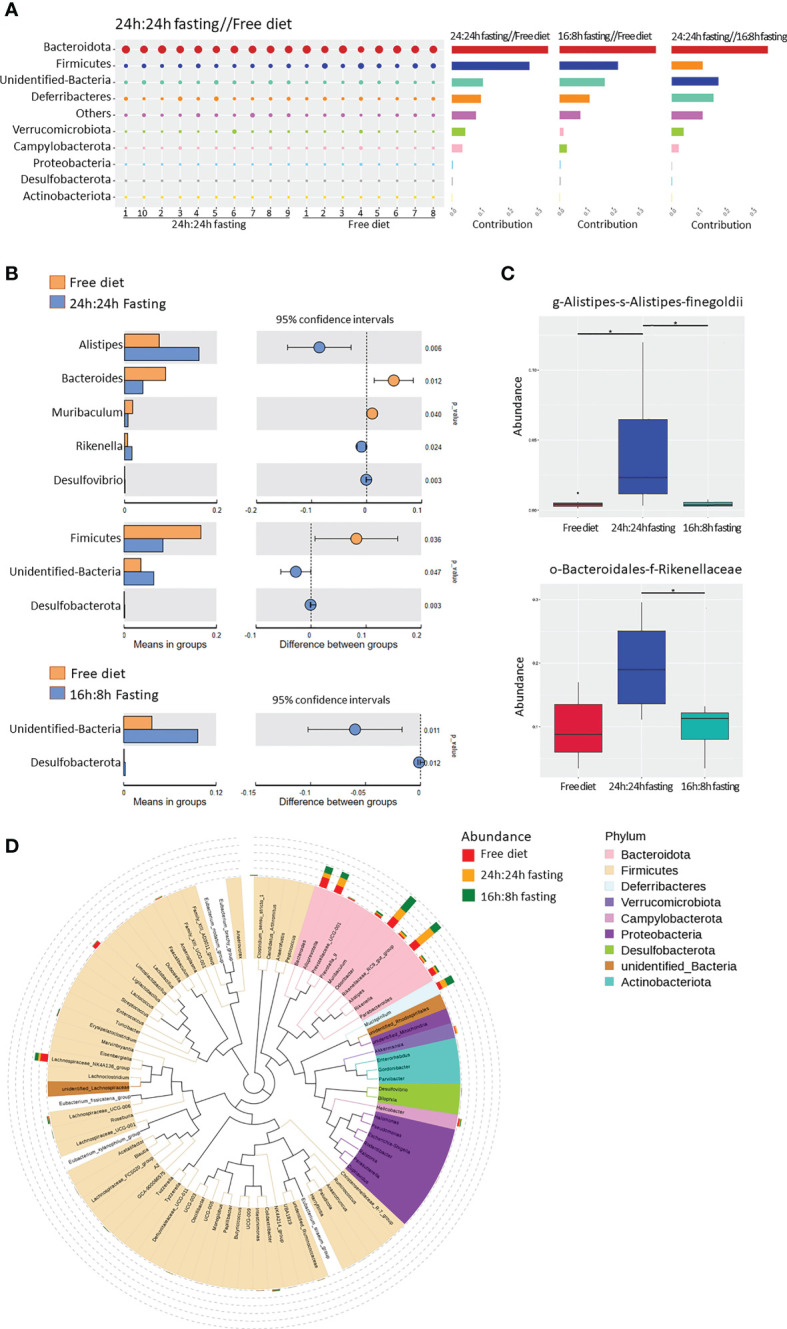
16s rRNA sequencing of gut microbiota, unique bacterial species analysis. **(A)** Similarity percentage. The bubble size represented the relative abundance of the corresponding species. **(B)** Species with significant differences between groups. The right figure showed the difference confidence between groups, two independent-sample t-test was used **(C)** Bacteria with the most significant differences between groups. Significance was analyzed by the Meta-Stat method: a hypothesis test was performed on species abundance between groups to obtain a p-value, which was then corrected by Benjamini-Hochberg false discovery rate to get the q-value. **(D)** Phylogenetic tree of genus-level species. The colors of branches and sectors indicated their corresponding phyla and the stacked bar plots on the outside of the fan ring indicated the information about the abundance distribution of the genus. All the Figures n=8,8,10 respectively in the FrD group, 16h: 8h IF group, 24h: 24h IF group. * p < 0.05.

The similarity percentage showed the top 10 contributors and their richness to the difference between the two groups. The top 1 contributor was *Bacteroidota*, thanks to their highest abundance and variety of species in the gut microbiota. The NO.2 contributor to the difference between FrD and 16/8F or 24/24F group was *Firmicutes*, which only ranked NO.4 between the 16/8F and 24/24F groups. *Firmicutes* appeared to be more similar within the two fasting groups than in the FrD group (**Figure 6A**). The abundance of the unidentified bacteria, *Alistipes* and *Rikenella* was significantly higher in the 24/24F group than in the FrD group. In contrast, the *Firmicutes*, *Bacteroides*, and *Muribaculum* were significantly lower in the 24/24F group compared to the FrD group. Only the richness of unidentified bacteria was significantly higher in the 16/8F group than in the FrD group (**Figure 6B**). The Meta-Stat box plots of the abundance showed that the abundance of *Alistipes* and *Rikenellaceae* were significantly higher in the 24/24F group than in other two groups (**Figure 6C**).

The phylogenetic tree showed the phylogenetic relationships at the genus level (**Figure 6D**). *Alistipes*, *Rikennella*, *Rikennellaceae-RC9-Gut group*, and *Odoribacter* were closely related in the evolutionary branches, and all of them increased in abundance in the fasted mice. *Alistipe* and *Odoribacter* had the highest abundance among all the species, while the *Alistipes* significantly increased in the 24h: 24h IF group, and *Odoribacter* increased in the 16h: 8h IF group. The high abundance of *Ligilactobacilus* and *Lachnospiraceae - Nk4a136-group* in the FrD group may be caused by outlier ‘mouse 4’, as mentioned in **Figure 5C**.

## 4 Discussion

In the current study, we showed that long-term IF was associated with a lower severity and incidence rate of FA symptoms, including rectal temperature decrease and diarrhea. IF reduced serum OVA-sIgE and -sIgG1 levels and attenuated Th2 cytokines expression in the serum and spleen of FA mice. IF decreased mast cell infiltration in the ileum mucosa and preserved the integrity of the intestinal epithelial barrier in FA mice. Moreover, IF had a profound effect on gut microbiota in FA mice.

OVA is an important culprit allergen in egg allergy (51). In this study, we used OVA as the allergen and aluminum hydroxide as the adjuvant to construct the FA mice model, as previously reported (48, 52–54). Significant hypothermic reactions and diarrhea, the two main symptoms that can be assessed to indicate the severity of FA, were observed in FA mice model (54–56). Both 16/8F and 24/24F groups demonstrated a reduction of FA symptoms. 24h:24h IF appeared to have more potent protective effects against FA since this mode of IF showed a more remarkable reduction in hypothermia and diarrhea as well as OVA-sIgE compared to 16h:8h IF. Scratching, bristled fur, laborious wheezing, abnormal immobility, and cyanosis were also reported as FA symptoms in the cholera toxin adjuvanted mice model (57), which were not observed in our study.

Two pathways have been suggested to induce allergic reactions in the FA mice model, the classical pathway mediated by IgE, FcϵRI, mast cells, histamine, and platelet-activating factor (PAF), and the alternative pathway mediated by IgG, FcγRIII, macrophages, and PAF (55). In humans, most food-induced systemic anaphylaxis is mediated by IgE, while IgG acts primarily as potential competitor of IgE for the binding of allergens (58). Due to the lack of IgG4 isotype (59–61), only total and OVA-specific IgG1 can be detected in the FA mice model. Elevated OVA-sIgG1 has been observed in FA mice model (54, 62, 63). We observed a similar reduction of OVA-sIgE and sIgG1 after IF when compared to a free diet. IgG1 expressing B cells in mice may be subjected to an antibody class switch in the presence of IL-4 and act as the primary source of IgE (64).

The duodenum and ileum intestine fluff can be damaged and eroded in the FA model. Previous studies showed that food allergens can induce gap expansion as well as tight junction damage in intestinal villus epithelial cells (65). We observed a similar structure change of ileum villi in all three groups of FA mice. In addition, increased mucosal mast cells were observed in FA mice, which may contribute to the hypothermic reaction and diarrhea, as the numbers of mast cells correlate with gastrointestinal anaphylaxis manifested as diarrhea (66). The FrD group had a higher incidence rate and a score of diarrheas compared to both fasting groups. Consistently, a higher number of mast cells was observed in the mucosa of the small intestine of FrD mice compared to both fasting mice. Thus, IF may relieve diarrhea by decreasing mast cell infiltration in the ileum. Additionally, decreased IgE antibodies in IF mice may also contribute to the relief of FA symptoms by suppressing the function of mast cells (67).

Intestinal epithelial barrier dysfunction may promote FA, and vice versa (65, 68). Allergens, certain kinds of bacteria, fungi, viruses, laundry and dishwasher detergents, household cleaners, surfactants, enzymes and emulsifiers in processed food, cigarette smoke, particulate matter, diesel exhaust, ozone, nanoparticles, and microplastics all may disrupt the epithelial barrier, reduce the defense function of the epidermal against allergens and results in a skewing of type 2 immune response (69, 70). Moreover, the development of leaky epithelial barriers can lead to microbial dysbiosis and ectopia (69). The expression of ZO-1, one of the compositions of tight junctions (TJs), was more extensive in the fasting mice than in FrD mice. Thus, IF appeared to be able to preserve the integrity of the intestinal barrier and prevent gut leakage (70).

Fasting was proved to blunt the Th2 cell response and airway epithelial cell cytokine production in steroid-naive asthmatics (71). This is consistent with our study which showed that IF could attenuate Th2 response and restore Th1/Th2 balance. The mRNA expression level of GATA3, the Th2 cytokine transcriptional factor, was also downregulated by IF, although without statistical difference. These data suggest that IF may suppress Th2 inflammation involved in FA by regulating GATA3 expression beyond the transcriptional level such as posttranslational modifications (72).

Previous studies indicate that gut microbiota dysbiosis may precede the development of FA, as shown in the FA mice model (12–14, 73). We observed an effect of IF on the gut microbiota of FA mice. *Alistipes* and *Rikenella* were significantly enriched in the 24/24IF mice compared to the other two groups of mice. And *Odoribacter* enriched in 16/8IF mice although lack of statistical difference. All of those three bacteria are bile resistant bacteria, and enriched in the ileocecal region. *Alistipes* could produce indol, succinic acid, and a small amount of acetic acid and propionic acid (74, 75). *Rikenella* produce mainly propionic acid and succinic acid, especially isomethyl branched acid form (74). *Rikenella* deficiency is associated with premature aging (76), as it was a key driver associated with long-term development of midlife gut microbiota (77). *Odoribacter* contributes to the increase of SCFAs (major acetic acid, propionic acid, and succinic acid), it also produces hydrogen and hydrogen sulfide (78). *Odoribacter splanchnicus* protect against colitis and colon cancer by inducing intestinal Th17 cells (79), and showing an anti-inflammatory activity by inducing IL-10 production (80).

The protective effect of SCFA on allergy has been addressed in a few studies. Propionate and acetate have been shown to induce the activation of P38- and ERK- MAPK on the intestinal epithelium, dendritic cells, and Tregs, which then promotes intestinal tolerance. Butyrate affects intestinal CD103^+^DC by stimulating the cell surface receptor GPR109a, to trigger the proliferation of mucosal Treg in mesenteric lymph nodes. In addition, SCFA can regulate the epigenetics of Treg to enhance its expansion or protect Foxp3 protein from degradation (81). In addition, SCFAs appear to maintain intestinal barrier function by stimulating the synthesis of antimicrobial peptides and IL-18 (82). Butyrate induces the expression of genes encoding TJs components and protein reassembly through the activation of transcription factors such as signal transducer and activator of transcription (STAT) 3 and specificity protein 1 (SP1). Propionate could reduce claudin-2 through STAT3 activation and histone deacetylases (HDACs) inhibition (83). Butyrate affects the O_2_ levels in epithelium resulting in activation of the oxygen sensor hypoxia-inducible factor (HIF), which is a transcription factor important to coordinating gut integrity (84).

However, it is necessary to confirm the potential role of *Alistipes*, *Rikenella*, and *Odoribacter* in the protection effect of IF against FA by using fecal microbiota transplantation.

The epithelial barrier could be protected by many commensal bacteria. *C. prevotella* could improve glucose homeostasis, mild inflammation, and intestinal epithelial permeability (85). *Bacteroides vulgatus* and *Bacteroides dorei* have been proven to increase ZO-1 expression and improve epithelial barrier function (86 **)**. *Akkermansia muciniphila* improves the integrity of the intestinal barrier directly by promoting the production of mucin and indirectly by interacting with other bacteria and is also associated with the reduction of low-grade inflammation (87).

Consistently, IF could increase the diversity of gut microbiota and improve the richness of *Lactobacillus*, *Prevotella*, and *Bacteroidaceae* in autoimmune diseases (44). In patients with metabolic syndrome, IF could increase the abundance of *Ruminococcaceae* and *Roseburia*, although it might not increase the overall diversity of gut microbiota (88). In addition, IF increased the OTU abundance of *Firmicutes*, while decreasing the OUT abundance of most other phyla in obesity (38). Another study in obesity reported that IF can increase the microbiome alpha diversity, and improve *Lactobacillus* and butyrate-producing *Odoribacter* while decreasing *Enterococcus*, *Streptococcus*, and unknown *Enterococcaceae (* 70).

The effect of IF on metabolism is also worth investigating. IF can reduce the production of energy and free radicals by mitochondrial, and promote the production of ketones, which then reduce low-density lipoprotein and increase high-density lipoprotein (89). Mitochondrial metabolism and oxidative phosphorylation are associated with Tregs differentiation (90), and glycolysis dysfunction of T cells has been observed in allergic diseases (91). Whether IF could protect FA by affecting T cell aerobic glycolysis or free radical production needs to be further investigated with metabolomic assays. In addition, IF may induce autophagy due to nutrient starvation, promote the degradation of claudin-2 through clathrin-mediated endocytosis, and then enhance the intestinal epithelial tight junction barrier.

We investigated two regimens of IF, 16h:8h and 24h:24h diet, which have been studied previously (92). It is not yet possible to make any clear conclusions about whether one IF regimen is superior to another, as it is rare to compare two IF regimens in the same study (93). It is worth mentioning that even a lower meal frequency and smaller meal sizes may have some effects as IF has (94). In our study, 24h: 24h IF had a more significant effect than 16h: 8h IF on protection against FA. Potentially, 8h eating for natural daytime conflicted with the habits and the circadian rhythm of mice (95). Such a disadvantage is not present in humans, as the 16h: 8h diet requires relatively minor lifestyle changes (95).

A previous study demonstrated that temporary fasting for 36 h aggravated FA. This kind of short-term fasting was associated with the depletion of immune cells and thus attenuated antigen-specific IgA response and oral tolerance (46). In our study, long-term fasting was performed at 16h: 8h or 24h:24h fasting-refeeding rhythm and continued for 28 days before being sensitized with OVA. Moreover, long-term fasting may potently remodel the gut microbiota, which may not be achieved by short time fasting. Our study represents the first to investigate the effect of long-term IF on FA in a murine model.

## 5 Limitations

This study confirmed that IF could protect FA and remodel gut microbiota, but did not implement fecal transplantation from IF mice or specific bacteria transplantation such as *Alistipes* and *Rikenella* to study the effect on FA directly, which needs further research. It should be noticed that this study was performed in mice maintained in an SPF environment, thus, the abundance and diversity of gut microbiota are different from that of the open environment. Mice in fasting groups were more likely to intake poplar fiber and feces due to starvation, and this behavior may have a regulating effect on the gut microbiome. The addition of screen mesh could prevent mice from intaking bedding material. Furthermore, it is difficult for the 24h:24h fasting protocol to be accepted and implemented by most people, whereas the 16h:8h fasting model may be more plausible.

## 6 Conclusion

Long-term IF may attenuate Th2 inflammation, maintain the integrity of the intestinal epithelial barrier, and protect FA *via* regulating gut microbiota in mice models.

## Data availability statement

The datasets presented in this study can be found in online repositories. The names of the repository/repositories and accession number(s) can be found below: https://cloud.189.cn/t/ARb2qmIBnqim, password mqs0.

## Ethics statement

The animal study was reviewed and approved by Animal Ethics Committee of Zhongnan Hospital of Wuhan University.

## Author contributions

Funding Acquisition: G-HL, Y-DG; Concept & Design: Y-DG, R-XM, X-LZ; Experiment perform & Data Analyze: R-XM, J-QH, WF, JZ, CC, C-CW, S-QQ; Manuscript Original Draft: R-XM; Manuscript – Review & Editing: Y-DG. All authors contributed to the article and approved the submitted version.
