# Supplementary material for: Intermittent fasting protects against food allergy in a murine model via regulating gut microbiota
Source: Front Immunol. 2023 May 9;14:1167562. doi: 10.3389/fimmu.2023.1167562 (PMC10205017; doi:10.3389/fimmu.2023.1167562)
Supplement: Supplementary file 1 [file DataSheet_1.docx]

Supplementary Material

Intermittent fasting protects against food allergy in a murine model via regulating gut microbiota

Ru-xue Ma*, Jia-qian Hu, Wei Fu, Jian Zhong, Can Cao, Chang-chang Wang, Shi-quan Qi, Xiao-Lian Zhang, Guang-hui Liu*, Ya-dong Gao*

*** Correspondence:** Ya-dong Gao: [gaoyadong@whu.edu.cn](mailto:gaoyadong@whu.edu.cn)；Guang-hui Liu：[ghliu-3488@163.com](mailto:ghliu-3488@163.com).

# Supplementary Tables

# Table S1 Primers for RT-PCR.

| Genes | Forward Sequence | Reverse Sequence |
| --- | --- | --- |
| T-bet | AACCGCTTATATGTCCACCCA | CTTGTTGTTGGTGAGCTTTAGC |
| GATA3 | AAGCTCAGTATCCGCTGACG | GTTTCCGTAGTAGGACGGGAC |
| FoxP3 | CACCTATGCCACCCTTATCCG | CATGCGAGTAAACCAATGGTAGA |
| RORγT | GACCCACACCTCACAAATTGA | AGTAGGCCACATTACACTGCT |
| IL-4 | ATCATCGGCATTTTGAACGAGG | TGCAGCTCCATGAGAACACTA |
| IL-10 | GCTGGACAACATACTGCTAACC | ATTTCCGATAAGGCTTGGCAA |
| IFN-γ | ATGAACGCTACACACTGCATC | CCATCCTTTTGCCAGTTCCTC |
| TGF-β 1 | CTTCAATACGTCAGACATTCGGG | GTAACGCCAGGAATTGTTGCTA |
| IL-5 | GCAATGAGACGATGAGGCTTC | GCCCCTGAAAGATTTCTCCAATG |
| IL-13 | CCTGGCTCTTGCTTGCCTT | GGTCTTGTGTGATGTTGCTCA |

# Supplementary Figures

**
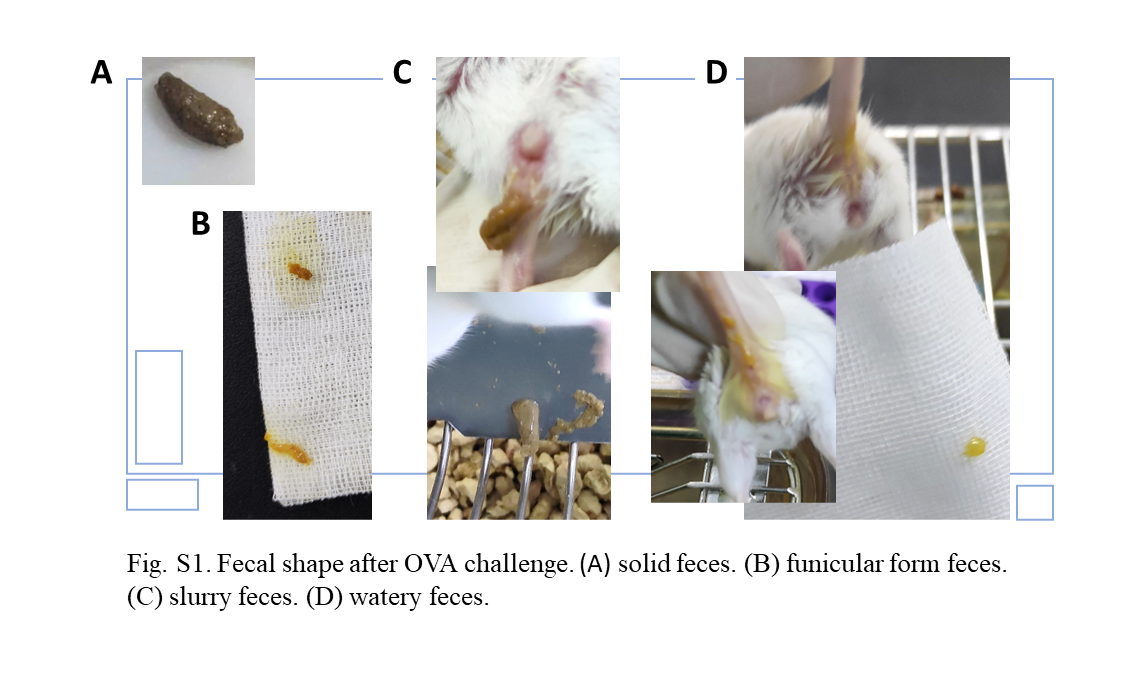
**

**Supplementary Figure 1.** The Fecal shape of mouse after OVA challenge. (A) solid feces. (B) funicular form feces. (C) slurry feces. (D) watery feces.


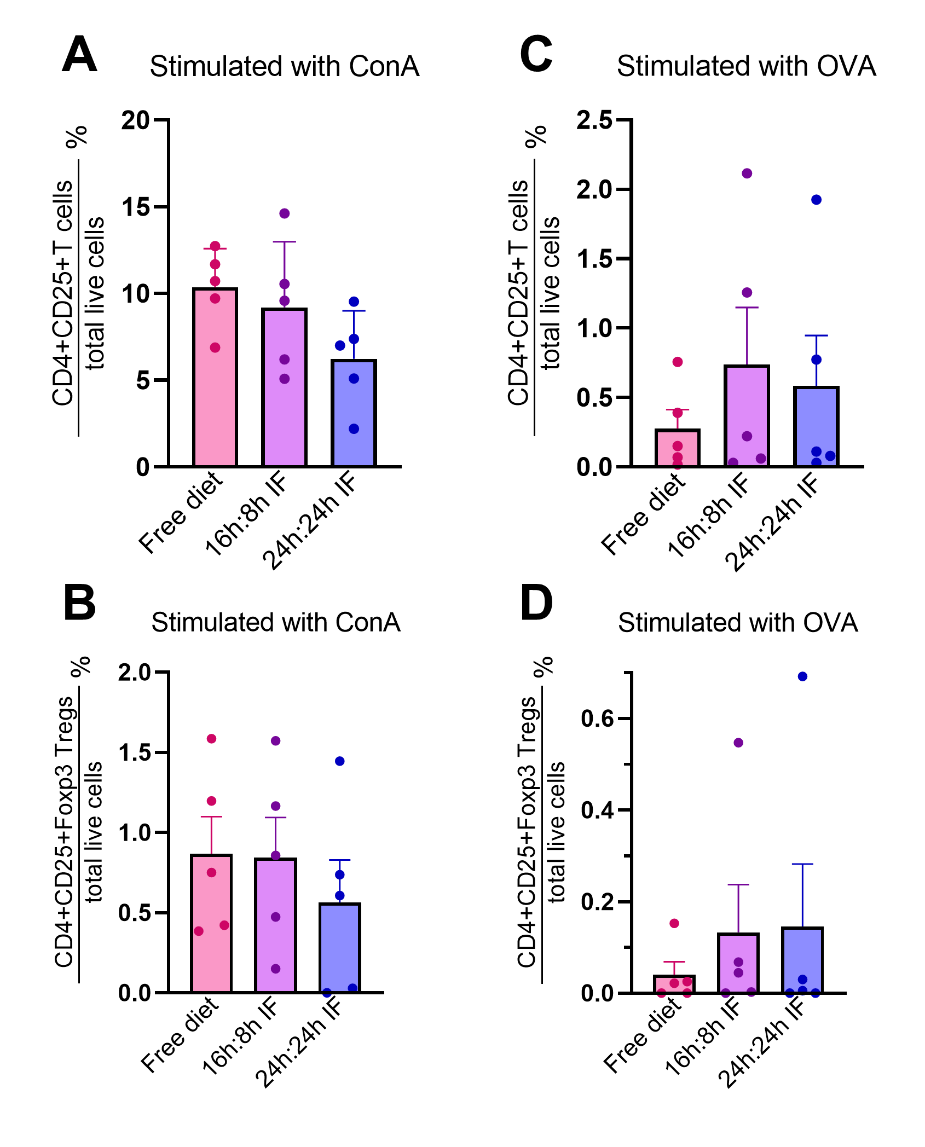


**Supplementary Figure 2.** **The proportion of Tregs in total cells.** (A-B) Stimulated with con A. (A) the proportion of CD4+ CD25+ T cells; (B) the proportion of CD4+ CD25+ Foxp3+Tregs; (C-D) Stimulated with OVA. (A) the proportion of CD4+ CD25+ T cells; (D) the proportion of CD4+ CD25+ Foxp3+Tregs. n=5 in each group. The statistical significance was analyzed by the Mann-Whitney test.

**
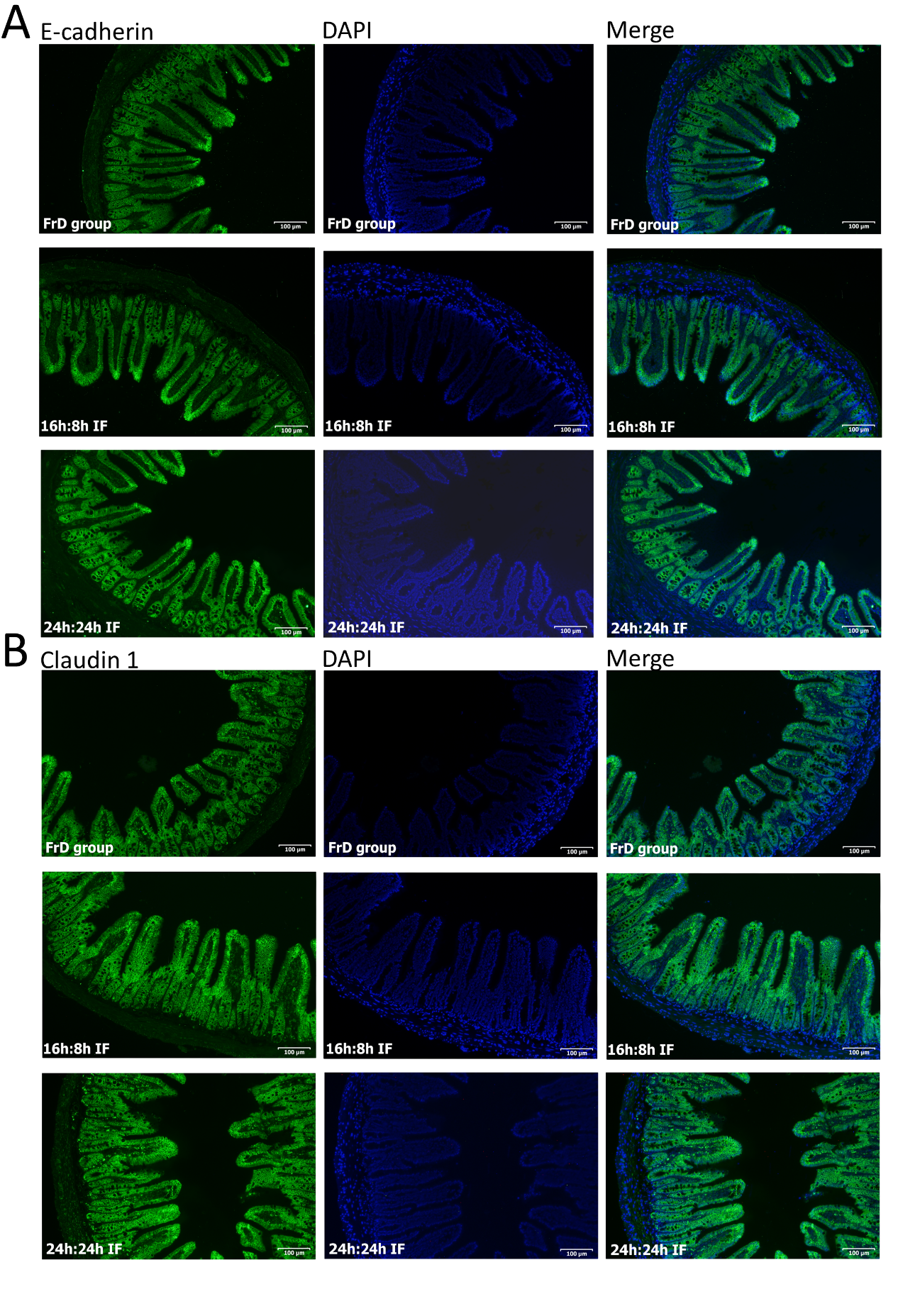
**

**Supplementary Figure 3.** Immunofluorescence micrographs. (A) E-cadherin. (B) Claudin 1.


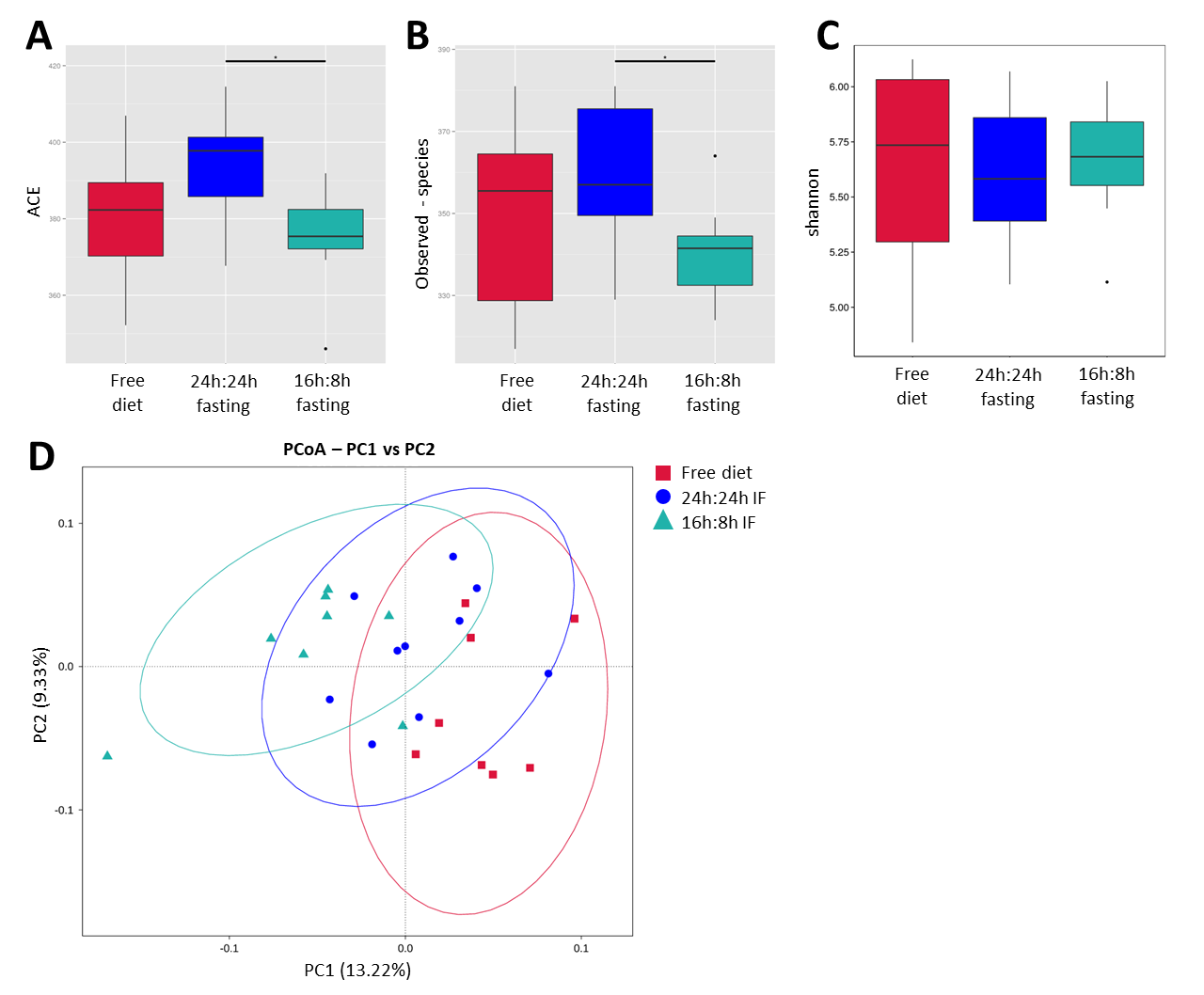


**Supplementary Figure 4.** **The difference in gut microbiota between groups.** (A) ACE diversity. (B) Observed – species diversity. (C) Shannon diversity. (D) Principal coordinates analysis (PCoA). n=8 in FrD group, n=8 in 16h: 8h IF group, and n=10 in 24h: 24h IF group. T-test and Wilcoxon rank-sum tests were performed when there were only 2 groups. Tukey and Wilcoxon rank-sum test were performed when the group was greater than 2. *p < 0.05.


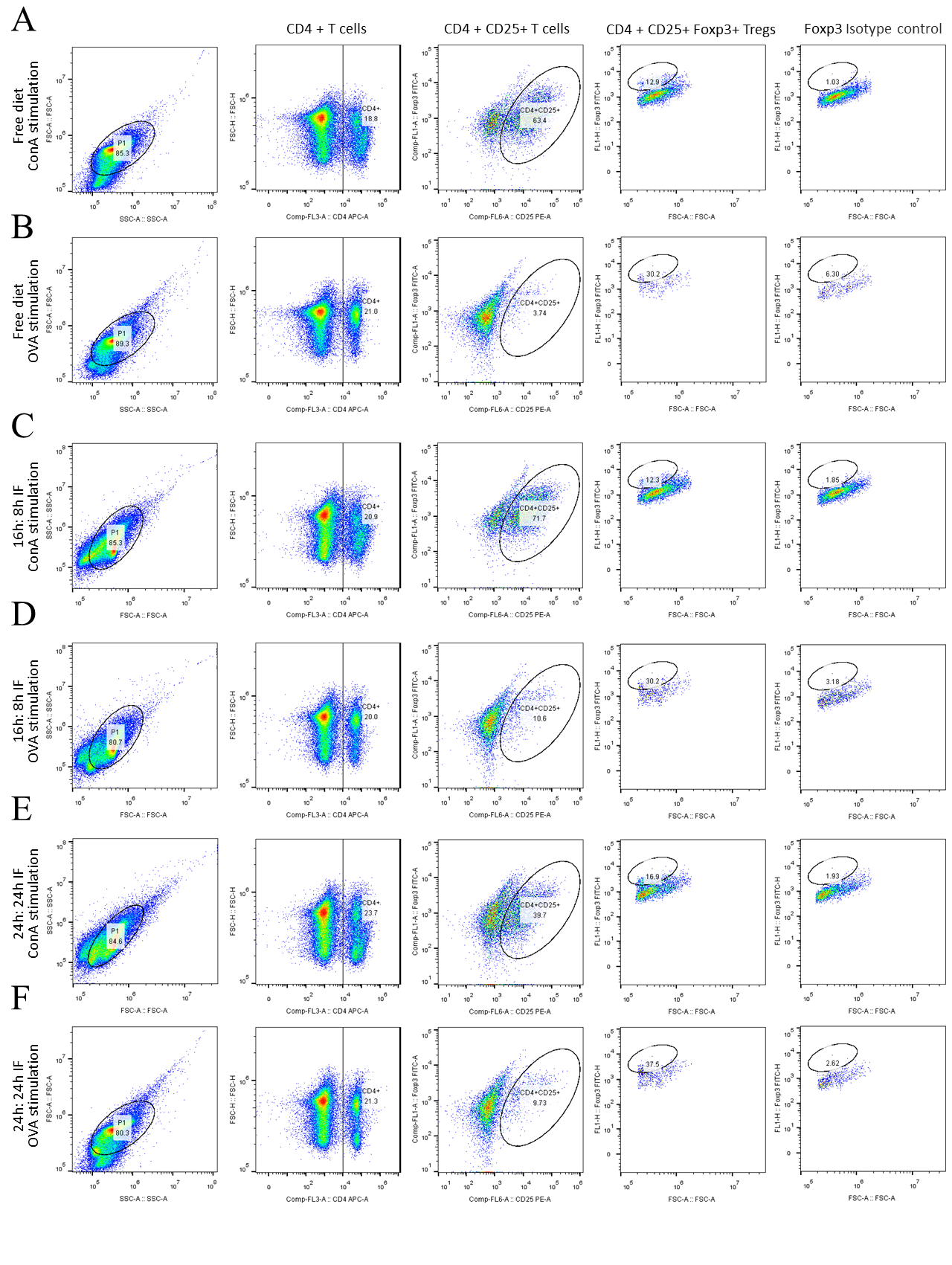


**Supplementary Figure 5.** CD4^+^ CD25^+^ Foxp3^+^ Tregs in *in vitro* cultured splenocytes from IF and free diet mice. (A). FrD mice group, stimulated with concanavalin A. (B). FrD mice group, stimulated with OVA. (C). 16h: 8h IF mice group, stimulated with concanavalin A. (D) 16h: 8h IF mice group, stimulated with OVA. (E) 24h :24h IF mice group, stimulated with concanavalin A. (F). 24h :24h IF mice group, stimulated with OVA.
